# Comprehensive bioinformatics and immunohistochemical analyses identify phosphoinositide metabolism and PNPLA7 as potential biomarkers in urological cancers

**DOI:** 10.1038/s41598-025-97118-9

**Published:** 2025-04-12

**Authors:** Yinhao Chen, Mingde Gao, Peng Chen, Amit Sharma, Maria Fitria Setiawan, Maria A. Gonzalez-Carmona, Xiaolin Wang, Ingo G. H. Schmidt-Wolf

**Affiliations:** 1 Department of Integrated Oncology, Center for Integrated Oncology (CIO), University Hospital of Bonn, Venusberg Campus 1, 53127 Bonn, Germany; 2 Department of Urology, Affiliated Tumor Hospital of Nantong University and Nantong Tumor Hospital, No. 30, Tongyang North Road, Tongzhou District, Nantong, 226361 Jiangsu People’s Republic of China; 3 Department of Internal Medicine I, University Hospital Bonn, Bonn, Germany

**Keywords:** Urological cancers, Phosphoinositide metabolism, Machine learning, Bioinformatics, Risk stratification, Urological cancer, Immune evasion, Molecular medicine, Cellular signalling networks, Data mining

## Abstract

Phosphoinositides (PI) and their metabolic enzymes are known to be involved in cellular processes associated with the hallmarks of cancer. The impact of PI metabolism-related components on urinary tract (or urological) cancers, however, remains unexplored. Considering this, herein, we performed a comprehensive bioinformatic analysis on clear cell renal carcinoma, bladder cancer, and adenocarcinoma of the prostate using public databases to investigate the relative contribution of PI metabolism in these clinical phenotypes. Primarily, we computed phosphoinositide metabolism scores to assess the associated biological processes and further enriched the analysis by predicting drug sensitivity, immune profiling, and risk stratification. Besides, we utilized single-cell RNA sequencing datasets to identify intercellular communication networks associated with PI metabolism in these cancer subtypes. Of interest, our analysis identified the PNPLA7 gene as a potential biomarker in urological cancers, which we validated using immunohistochemical evaluation of clinical samples. In conclusion, our study reveals that PI metabolism is a critical prognostic biomarker for urological cancers and may guide drug therapies, including immunosuppressants. Therefore, PNPLA7 could serve as a potential target and requires further attention.

**Supplementary Information:**

The online version contains supplementary material available at 10.1038/s41598-025-97118-9.

## Introduction

Urological cancers, including kidney, bladder, and prostate cancer, impose a substantial burden on global health^1–3^. Treatment modalities for these malignancies are diverse, ranging from surgical interventions, such as radical prostatectomy for urological cancers, to advanced therapeutic strategies^4–6^. Immunotherapy, particularly immune checkpoint inhibitors, has shown great promise in urological cancers, with PD-L1 expression serving as a valuable predictive biomarker^7^. In addition, targeted therapies, including monoclonal antibodies and tyrosine kinase inhibitors, play a crucial role in managing these neoplasms^8^. Despite these therapeutic advancements, significant challenges remain, particularly in bladder cancer, where high recurrence rates and progression to invasive disease are prevalent^9^. These issues underscore the need for continued research into novel therapeutic strategies and the identification of new biomarkers.

Phosphoinositides (PI) metabolism plays a critical role in numerous cellular functions, including lipid metabolism and signal transduction pathways^10^. Research has demonstrated that phosphoinositide 3-kinase (PI3K) inhibitors can modulate immune responses and induce metabolic alterations, underscoring the complex relationship between PI metabolism and immune regulation^11^. In cancer, dysregulation of phosphatidylinositol signaling pathways has been implicated in tumorigenesis and cancer progression^12^. The PI3K pathway, frequently altered in malignancies, has emerged as a key therapeutic target in urological cancers^13^. Overall, PI metabolism represents a multifaceted process that influences cellular functions, ranging from lipid regulation to immune modulation and cancer development. A deeper understanding of the molecular networks governing PI metabolism is essential for elucidating its broader implications in health and disease.

Although PI metabolism has been widely investigated, its comprehensive landscape in urological tumors remains inadequately characterized. This study offers a large-scale, systematic analysis of PI metabolism and the associated gene landscape in kidney renal clear cell carcinoma (KIRC), bladder cancer (BLCA), and prostate adenocarcinoma (PRAD) across multiple databases. PI metabolic activity was quantified using computational algorithms, and PI metabolism scores, along with the roles of key genes, were explored through multiple dimensions, including gene expression patterns, patient prognosis, single-nucleotide variants (SNVs), cancer stem cell properties, the tumor microenvironment, molecular subtypes, and drug sensitivity. Furthermore, the impact of PI metabolism at the cellular level was examined by applying these analyses to single-cell datasets, offering deeper insights into its role within the tumor microenvironment.

## Methods

### Data acquisition

Expression profiling data of urological tumors and normal tissues were retrieved from the UCSC Xena platform by downloading the pan-cancer dataset “EB++AdjustPANCAN_IlluminaHiSeq_RNASeqV2.geneExp.xena,” with values in log2(norm_value + 1). Phenotypic data, including tumor type, survival outcomes, and immune subtypes, were also obtained. Expression data were aligned with survival information, yielding 1997 samples. The gene list “REACTOME_PI_METABOLISM.v2023.2.Hs.gmt” was sourced from the Molecular Signatures Database to extract a PI metabolism-specific expression matrix. Pathway scoring, including PI metabolism, was conducted using single-sample gene set enrichment analysis (ssGSEA) via the “GSVA” package, followed by Z-score transformation for normalization^14^. SNV data, “mc3.v0.2.8.PUBLIC.maf.gz”, were also downloaded. Lastly, single-cell RNA sequencing datasets GSE207493, GSE135337, and GSE141445 were collected, comprising 19 KIRC, 7 BLCA, and 13 PRAD samples for detailed single-cell analysis.

### Screening for key genes via machine learning

In this study, we used three complementary machine learning algorithms, LASSO regression, random survival forest, and an XGBoost model, to identify prognostic genes associated with PI metabolism in a high-dimensional dataset integrating gene expression and survival data. First, we conducted LASSO regression with the “glmnet” package under tenfold cross-validation to optimize the penalty parameter and retain genes with non-zero coefficients^15^. In parallel, we implemented a random survival forest model using the “randomForestSRC” package to account for complex, non-linear interactions and derive variable importance (VIMP) scores, selecting the most prognostically relevant genes. Furthermore, we constructed an XGBoost model for 100 boosting iterations, leveraging its gradient-boosting framework to detect influential predictors and isolate the top 10 features based on importance rankings. We then generated Venn diagrams to highlight intersecting genes across these three methods, thus creating a more robust consensus set of candidate genes for subsequent validation. LASSO excels at sparsity-inducing feature selection in high-dimensional data while mitigating overfitting, random survival forest captures potential variable interactions via an ensemble-based approach, and XGBoost offers strong predictive performance and computational efficiency.

### Prognostic analysis

Survival metrics, including overall survival (OS), disease-specific survival (DSS), and progression-free interval (PFI), were screened for further analyses. To evaluate the prognostic significance of PI metabolism scores (PIMS) and key genes involved in PI metabolism (PIKG), Cox proportional hazards regression analysis and the Kaplan–Meier (KM) method were employed. Patients with urological tumors from TCGA were randomly divided into training and testing cohorts in a 7:3 ratio. Using the training cohort data, a PI metabolism-related risk stratification (PIMRRS) model for urological tumors was constructed through a stepwise approach involving univariate Cox regression analysis, LASSO regression, and multivariate regression analysis. The prognostic accuracy of the model was validated through KM analysis and univariate Cox regression. Additionally, a nomogram incorporating PIMRRS, age, gender, and cancer type was developed using multivariate regression analysis and the “rms” package. To further assess the model’s prognostic performance, receiver operating characteristic (ROC) curves, calibration plots, and decision curve analysis (DCA) plots were generated. The ROC curve was used to determine the area under the curve (AUC), with higher values indicating greater predictive efficiency. The calibration plot compared the actual outcomes to the ideal prediction line, allowing assessment of the model’s predictive accuracy. The DCA plot evaluated the net clinical benefit of the nomogram, determining whether its performance significantly exceeded that of other models across a range of decision thresholds.

### Mutation analysis

In this study, copy number variation (CNV) data was downloaded from the TCGA database. GISTICS2.0, which identifies significant amplifications and deletions across the set of patients, was used to analyze the CNV data^16^. After downloading the SNV data, both non-silent and silent mutations were counted for each sample. Mutation frequency for each cancer type was calculated as the ratio of samples with non-silent mutations to the total number of samples. If only silent mutations were detected in a sample without non-silent mutations, the mutation frequency was recorded as 0. Finally, the “maftools” R package was utilized to visually represent the SNV mutation landscape in a waterfall plot^17^, providing a clear overview of mutation patterns across the samples.

### Enrichment analysis

The enrichment analyses in this study primarily focused on Gene Ontology (GO) and Kyoto Encyclopedia of Genes and Genomes (KEGG) pathways^18,19^, conducted based on groupings derived from PIMS and the PIMRRS. Differential expression analysis was performed using the “limma” package as previously described^20^. GO and KEGG pathway enrichment was carried out using the “enrichGO” and “enrichKEGG” functions in the “clusterProfiler” package^21^. To ensure robustness, all analyses were filtered based on an adjusted *p* value threshold of less than 0.05.

### Immune microenvironment and tumor stemness

The stromal and immune cell infiltration scores for patients with urological cancers were calculated using the “estimate” package^22^, which provides three key metrics: StromalScore, ImmuneScore, and ESTIMATEScore. These scores quantify the degree of stromal cell infiltration, immune cell infiltration, and the overall proportion of non-tumor cells, respectively. Additionally, immune cell infiltration data for urological cancer samples were obtained from the TIMER 2.0 database^23^, using XCELL, TIMER, QUANTISEQ, MCPCOUNTER, EPIC, CIBERSORT-ABS, and CIBERSORT. For immune escape analysis, the expression matrix of urological tumors was uploaded to the tumor immune dysfunction and exclusion (TIDE) platform^24^. The downloaded results were screened for key sections, including TIDE, MSI, Dysfunction, and Exclusion. Then, the expression data for immune checkpoints were extracted from the previously generated whole expression matrix for further analysis. Moreover, tumor stemness scores, including DNA methylation-based (DNAss) and RNA expression-based (RNAss), were retrieved from the UCSC pan-cancer dataset. Comparative analysis of these metrics and correlation studies were conducted to explore the relationships between stromal/immune infiltration, immune escape mechanisms, tumor stemness, and the expression of immune checkpoints in urological tumors.

### Drug sensitivity

Anti-cancer drug sensitivity data from the genomics of drug sensitivity in cancer (GDSC)^25^ and CellMiner databases^26^ were utilized in this study. The IC50 values of the drugs were calculated using the calcPhenotype function from the “oncoPredict” R package^27^. Additionally, gene expression and drug susceptibility score data for 60 cancer cell lines (NCI-60) were downloaded from the CellMiner database. Clinically tested and FDA-approved drugs were selected for analysis, and any missing data were imputed using the Impute.knn function. Subsequent analyses included subgroup comparisons based on PIMS and correlation assessments between PIMS and drug sensitivity, aiming to explore the relationship between PI metabolism and drug sensitivity in urological tumors.

### Single cell analysis

In this single-cell transcriptomic study, we focused on the PI metabolism pathway. Quality control procedures followed established protocols from prior studies, using the “DoubletFinder” package to eliminate doublets and the “harmony” package for batch correction. We annotated the single-cell profiles with a custom computational pipeline, applying dimensionality reduction via UMAP and identifying cell types accordingly. Single-cell PI metabolism scores were calculated for each identified cell type using the “AddModuleScore” algorithm. Based on this stratification, we employed the “CellChat” package to infer and compare intercellular communication networks, particularly between epithelial tumor cell subtypes and other cell populations. Furthermore, receptor-ligand interaction analyses were conducted within these subgroups to uncover how PI metabolism influences cellular communication dynamics and identify potential discrepancies in signaling pathways. This provided insights into the role of PI metabolism in modulating tumor-immune interactions at the single-cell level.

### Immunohistochemistry

To verify the expression of key genes at the histological level, we collected a total of urological tumor samples for immunohistochemistry from the Affiliated Cancer Hospital of Nantong University, including 5 pairs of patient samples from BLCA, 6 pairs of patient samples from KIRC and 4 pairs of patient samples from PRAD. This study has been approved by Nantong Tumor Hospital Medical Ethics Committee. The oral consent was obtained from the patients for the use of their tissues in research. We deparaffinized and rehydrated paraffin-embedded tissue from KIRC patients. Following antigen retrieval using microwaves, 3% hydrogen peroxide was applied to inhibit endogenous peroxidase activity, followed by 10% goat serum for further blocking. Having incubated the PNPLA7 primary antibody (Invitrogen, 1:100) at 4 °C all night, the corresponding secondary antibody was applied and DAB stained after rewarming and rinsing. In preparation for microscopic observation, the sections were counterstained with hematoxylin, dehydrated, air-dried, and covered. Immunohistochemistry results were interpreted as follows: no apparent staining (0), light yellow staining (1), brownish-yellow staining (2), and brown staining (3). Staining extent scores were evaluated as follows: ≤ 10% (0), 10–25% (1), 25–50% (2), 50–75% (3), and > 75% (4). Multiplying the scores for intensity and extent yielded the final score. In all cases, two pathologists evaluated double-blind slides based on the intensity and extent scores.

### Statistical analyses

The bioinformatics analyses described above were conducted using R version 4.3.1. For KM survival analysis, the median value was used as the cutoff point to stratify the groups. Comparisons between two groups of samples were made using the Wilcoxon test, while comparisons involving more than two groups were performed using the Kruskal–Wallis test. Correlation analyses were conducted using the Spearman test, with *p* < 0.05 considered as the threshold for statistical significance in all analyses.

## Results

### Clustering of pan-urological tumors based on PI metabolic pathway

**Fig. 1:**
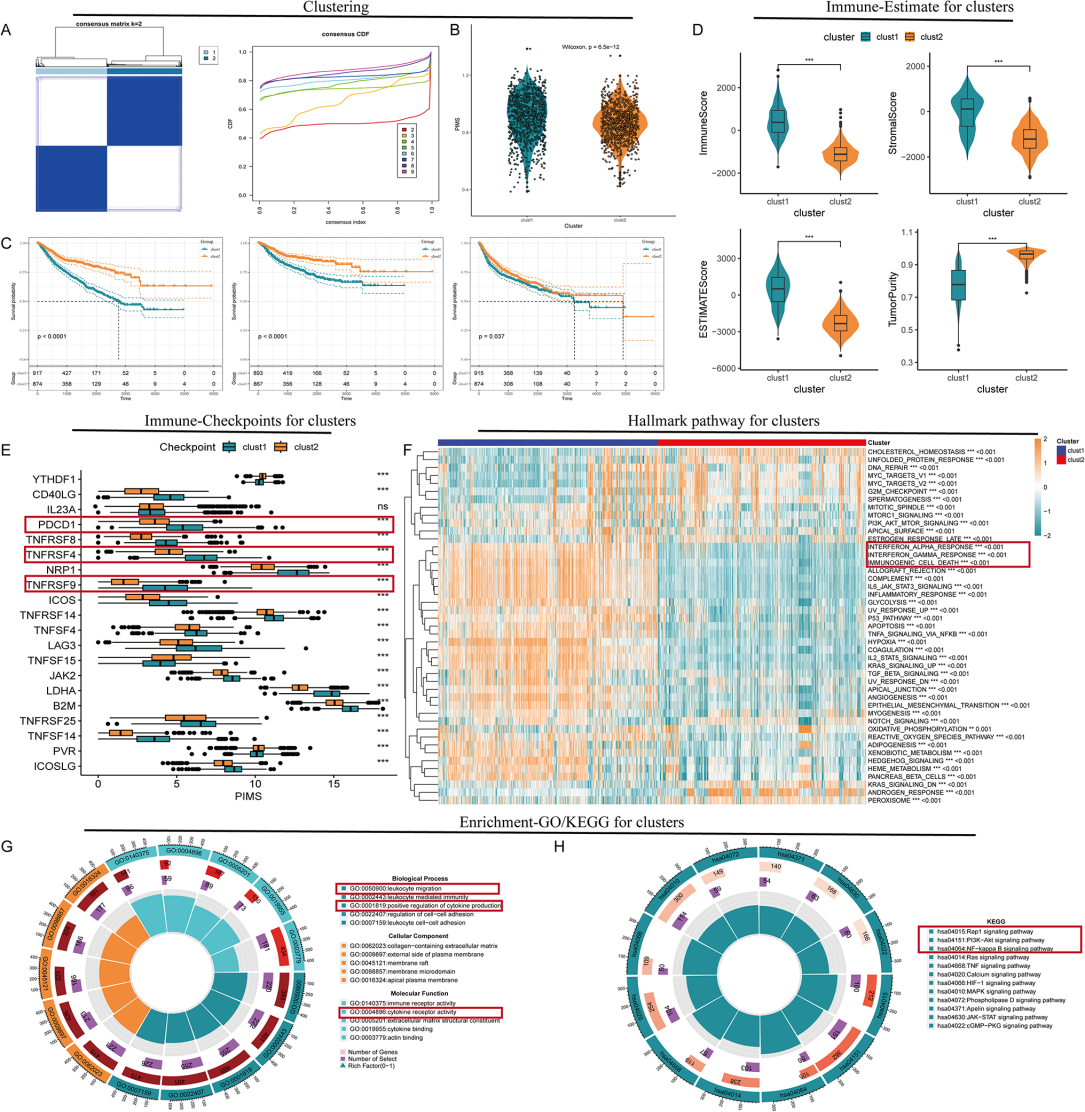
Clustering analysis based on PI metabolism in urological cancers. (**A**) Consensus matrix for k = 2 reveals two distinct clusters (Cluster 1 and Cluster 2), indicating robust partitioning in the dataset. The heatmap displays a dichotomy between the clusters, with deep blue indicating areas of high similarity. (**B**) Violin plots depict a significant difference in PIMS between the two clusters, with Cluster 1 exhibiting higher PIMS (*p* = 6.5e−12). The width of the violin plots represents the distribution of values. (**C**) Kaplan–Meier survival curves for OS, DSS, and PFI show significantly worse survival outcomes in Cluster 1 compared to Cluster 2 (all *p* < 0.05). The dashed lines represent the confidence intervals of survival probability. (**D**) Violin plots of the immune, stromal, and ESTIMATE scores indicate higher immune infiltration and stromal content in Cluster 1 (*p* < 0.001). (**E**) Box plots comparing the expression levels of key immune checkpoint regulators (e.g., PDCD1, TNFRSF9) across the clusters, with higher levels observed in Cluster 1. (**F**) ssGSEA pathway analysis reveals increased activation of immune-related pathways, including interferon-alpha and gamma responses, in Cluster 1. The color gradient represents the magnitude of enrichment. (**G**) GO enrichment analysis indicates that differentially expressed genes in Cluster 1 are enriched in immune-related biological processes such as cytokine receptor activity and leukocyte migration. (**H**) KEGG pathway analysis shows significant enrichment in pathways related to cytokine-cytokine receptor interaction and Rap1 signaling in Cluster 1.

The clustering analysis of urological cancers based on PI metabolism identified two distinct groups (Fig. 1A). The consensus matrix for k = 2 demonstrated a clear dichotomy, underscoring robust dataset partitioning. Cluster 1 displayed significantly elevated PIMS compared to Cluster 2 (*p* = 6.5e−12, Fig. 1B). KM analysis revealed that Cluster 1 had poorer outcomes in OS, DSS, and PFI (all *p* < 0.05, Fig. 1C). Further analysis of the tumor microenvironment indicated that Cluster 1 exhibited higher immune, stromal, and ESTIMATE scores (*p* < 0.001, Fig. 1D). Furthermore, immune checkpoint regulators PDCD1, TNFRSF9, and TNFRSF4 were notably upregulated in Cluster 1 (Fig. 1E). The ssGSEA analysis revealed that Cluster 1 was predominantly characterized by the activation of immune-related pathways, including interferon-alpha response, interferon-gamma response, and immunogenic cell death (Fig. 1F). GO enrichment analysis showed that differentially expressed genes were enriched in immune-related functions, such as cytokine receptor activity, immune receptor activity, leukocyte migration, and cytokine production regulation (Fig. 1G). KEGG pathway analysis highlighted significant activity in pathways related to Rap1 signaling, PI3K/AKT signaling, and NF/kappa B signaling pathways (Fig. 1H).

### The landscape of PIMS in urological cancers

**Fig. 2:**
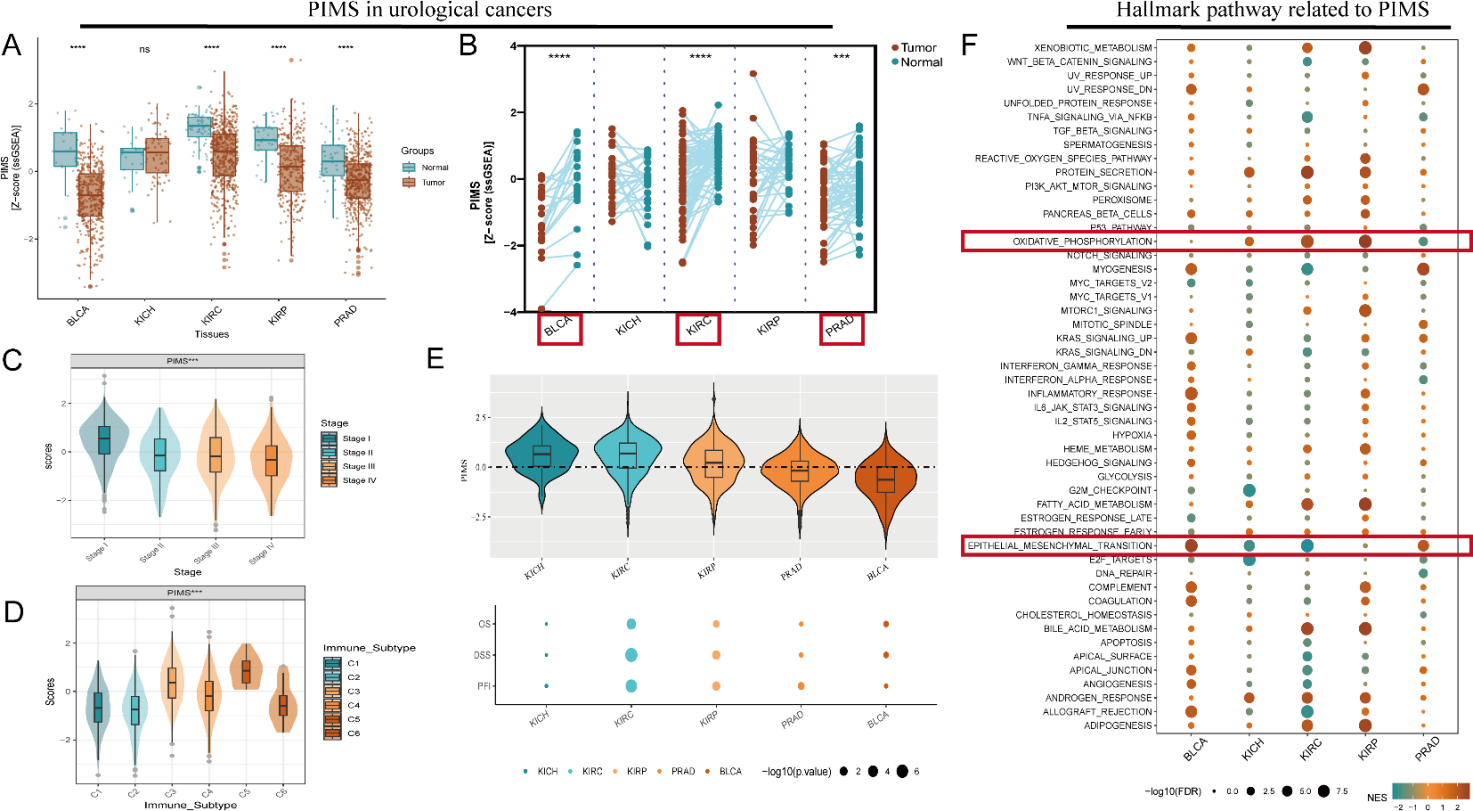
The landscape of PIMS at the bulk level. (**A**) Boxplots of PIMS in bladder cancer (BLCA), PRAD, kidney renal clear cell carcinoma (KIRC), and kidney renal papillary cell carcinoma (KIRP) tissues show statistically significant lower PIMS in tumors compared to adjacent normal tissues (*p* < 0.0001). The central line indicates the median. (**B**) Paired analysis between tumor and adjacent normal tissues for BLCA, PRAD, and KIRC confirms a consistent downward trend in tumor tissues (*p* < 0.0001). (**C**) Analysis of PIMS scores across different disease stages shows a decreasing trend as the disease advances. (**D**) Violin plots indicated a higher PIMS for subtype C5 compared to others. Violin plots and boxplots represent score distributions, with the black lines in the center denoting the median score at each stage. (**E**) Univariate Cox regression showed that PIMS was significantly associated with OS, DSS, and PFI in KIRC and KIRP (*p* < 0.05). Each dot represents the magnitude and significance of hazard ratios, and dot size correlates with statistical significance. (**F**) Hallmark pathway analysis shows that oxidative phosphorylation is significantly associated with PIMS in urological cancers. In the above analyses, significance levels are indicated by ns (non-significant), *(*p* < 0.05), ***(*p* < 0.001), and ****(*p* < 0.0001).

In BLCA, PRAD, KIRC, and kidney renal papillary cell carcinoma (KIRP) tissues, tumors exhibited significantly lower PIMS levels compared to normal tissues (*p* < 0.0001, Fig. 2A). Paired analyses of the tumor and adjacent normal tissues confirmed these trends in BLCA, PRAD, and KIRC (*p* < 0.001, Fig. 2B). A decreasing PIMS trend was also observed with advancing disease stages (*p* < 0.001, Fig. 2C). Immune subtype-related analysis further demonstrated that subtype C5 showed significantly higher scores, implying stronger immunosuppressive effects (*p* < 0.001, Fig. 2D). Univariate Cox regression analyses revealed that PIMS were significantly associated with OS, DSS, and PFI in KIRC, as well as DSS and PFI in KIRP (Fig. 2E). These findings underscore the potential of PIMS as a prognostic biomarker. Additionally, pathway differentiation analyses showed a significant correlation between PIMS and oxidative phosphorylation across cancer types, except for BLCA. Importantly, the epithelial-mesenchymal transition (EMT) pathway was notably regulated in BLCA, kidney chromophobe (KICH), KIRC, and PRAD (Fig. 2F), suggesting a potential link between PI metabolism and key oncogenic pathways.

**Fig. 3:**
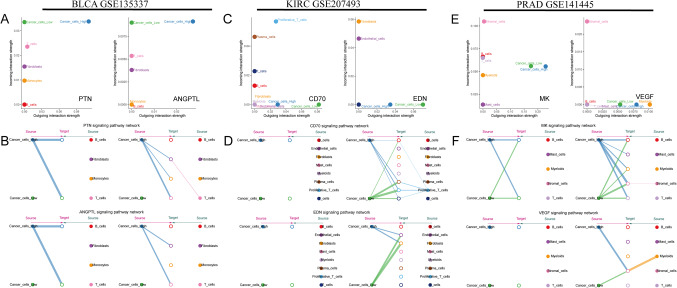
The landscape of PIMS at single-cell level. (**A**,**B**) In BLCA, high-PIMS cancer cells actively contributed to PTN and ANGPTL pathways. (**C**,**D**) In KIRC, low-PIMS cancer cells engaged in CD70 and EDN pathways. (**E**,**F**) In PRAD, high-PIMS cells activated MK and VEGF pathways.

A thorough examination of single-cell data from BLCA tissues identified distinct cellular populations marked by specific cell markers (Supplementary Fig. S1). Quantitative analysis revealed an elevated level of cancer cells. Cell communication analysis indicated similar outgoing signaling patterns among high-PIMS and low-PIMS cancer cells, as well as B cells, while cancer cells, fibroblasts, and T cells exhibited similar incoming communication patterns. Notably, high-PIMS cancer cells contributed more to outgoing communication than low-PIMS cells, particularly in the PTN and ANGPTL pathway networks (Fig. 3A). High-PIMS cancer cells also showed increased interaction with fibroblasts and T cells within these communication networks (Fig. 3B). The same approach was applied to KIRC and PRAD respectively, finding that clustering and annotation of different cell populations (Supplementary Figs. S2 and S3) with significant markers revealed PIMS variations across cell types, particularly in cancer cells. Similar outgoing and incoming communication patterns were observed between high-PIMS and low-PIMS cancer cells in KIRC and PRAD, with outgoing communication being predominant. In KIRC, the CD70 and EDN pathway networks were more prominent in low-PIMS cancer cells, while the MK and VEGF pathways were more active in high-PIMS cancer cells in PRAD (Fig. 3C,E). Immune cells, such as T cells, plasma cells, and myeloid cells, played a central role in these interactions (Fig. 3D,F). Overall, single-cell RNA sequencing revealed distinct cellular populations and pathway networks in urological cancers, which may be caused by PI metabolism.

### The landscape of PIMS in tumor microenvironment

**Fig. 4:**
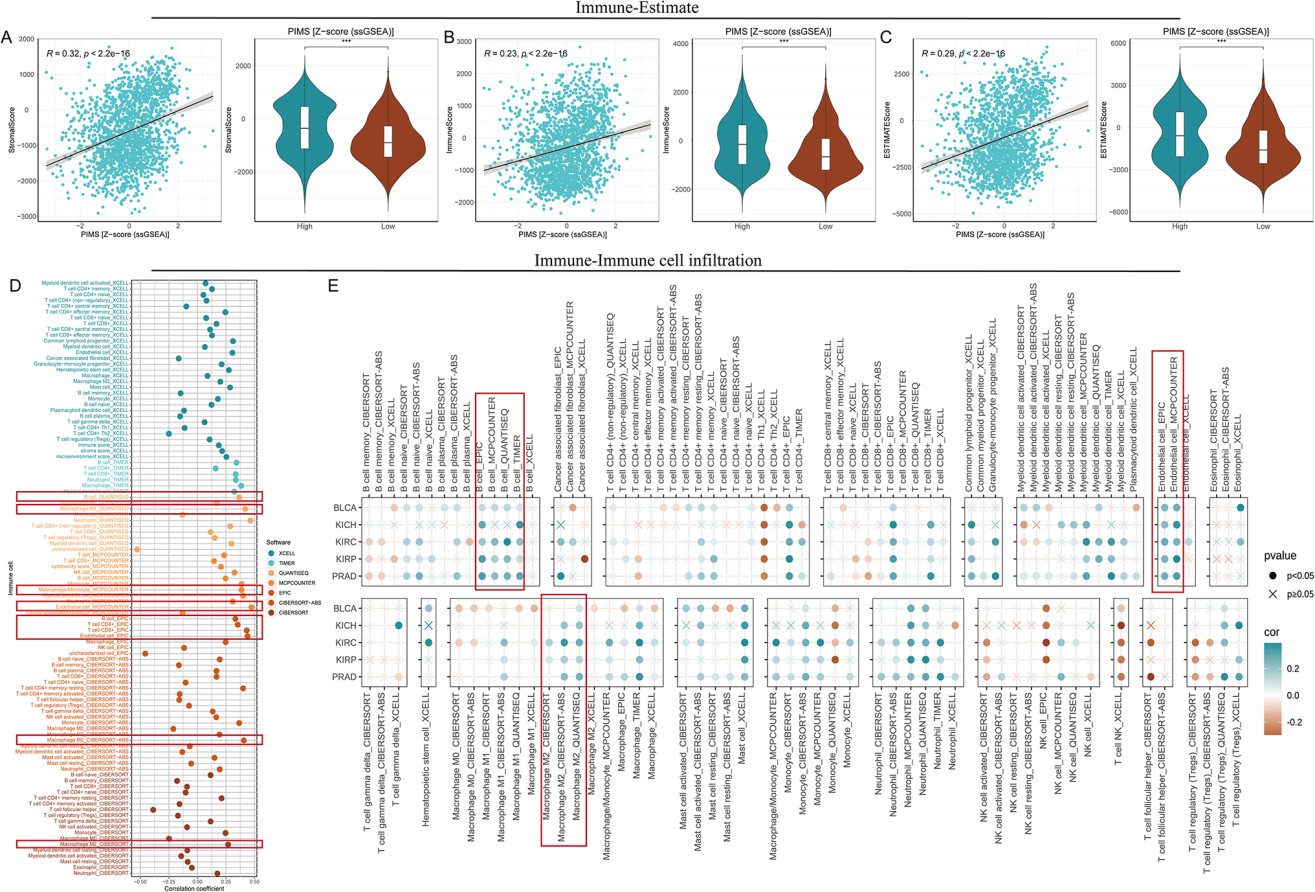
Correlation of PIMS with tumor microenvironment and immune cell infiltration. (**A**–**C**) The scatter plot shows significant positive correlations between PIMS and StromalScore, ImmuneScore as well as non-tumor score. The violin plot compares the three scores between high and low PIMS groups. (**D**) The correlation heatmap presents the association between PIMS and various immune cell infiltrates across five distinct algorithms. Significant positive correlations are observed with PIMS, such as M2 macrophage and B cells using QUANTISEQ, and endothelial cells in MCPCOUNTER. (**E**) A more detailed breakdown of immune infiltration by cancer type is shown using a bubble plot. Shades of color represent the magnitude of the correlation, and color indicates the direction (blue for positive, brown for negative).

In Fig. 4A, a significant positive correlation was identified between PIMS and StromalScore (R = 0.32, *p* < 2.2e−16), indicating that higher PIMS are associated with elevated stromal scores. Weaker correlations were observed in Fig. 4B (R = 0.23, *p* < 2.2e−16) and Fig. 4C (R = 0.29, *p* < 2.2e−16), corresponding to immune score and non-tumor score, respectively. Additionally, both StromalScore and ImmuneScore were elevated in high-PIMS patients with KIRC and PRAD, but notably lower in BLCA (Supplementary Fig. S4). Follow-up correlation analysis confirmed these patterns. The correlation heatmap in Fig. 4D reveals a variable relationship between PIMS and immune cell infiltration across the different algorithms. Several significant positive correlations with PIMS were observed, including M2 macrophages and B cells in QUANTISEQ, Macrophages/Monocytes and endothelial cells in MCPCOUNTER, and CD4+ T cells, CD8+ T cells, Endothelial cells, and B cells in EPIC. Further analyses of specific urological cancers showed that M2 macrophages, B cells, and Endothelial cell infiltration were positively correlated with PIMS in all cases, except for XCELL (Fig. 4E). These findings underscore the potential of PIMS in predicting cell infiltration patterns of immune cells, such as M2 macrophages and B cells.

### Immunotherapeutic response

**Fig. 5:**
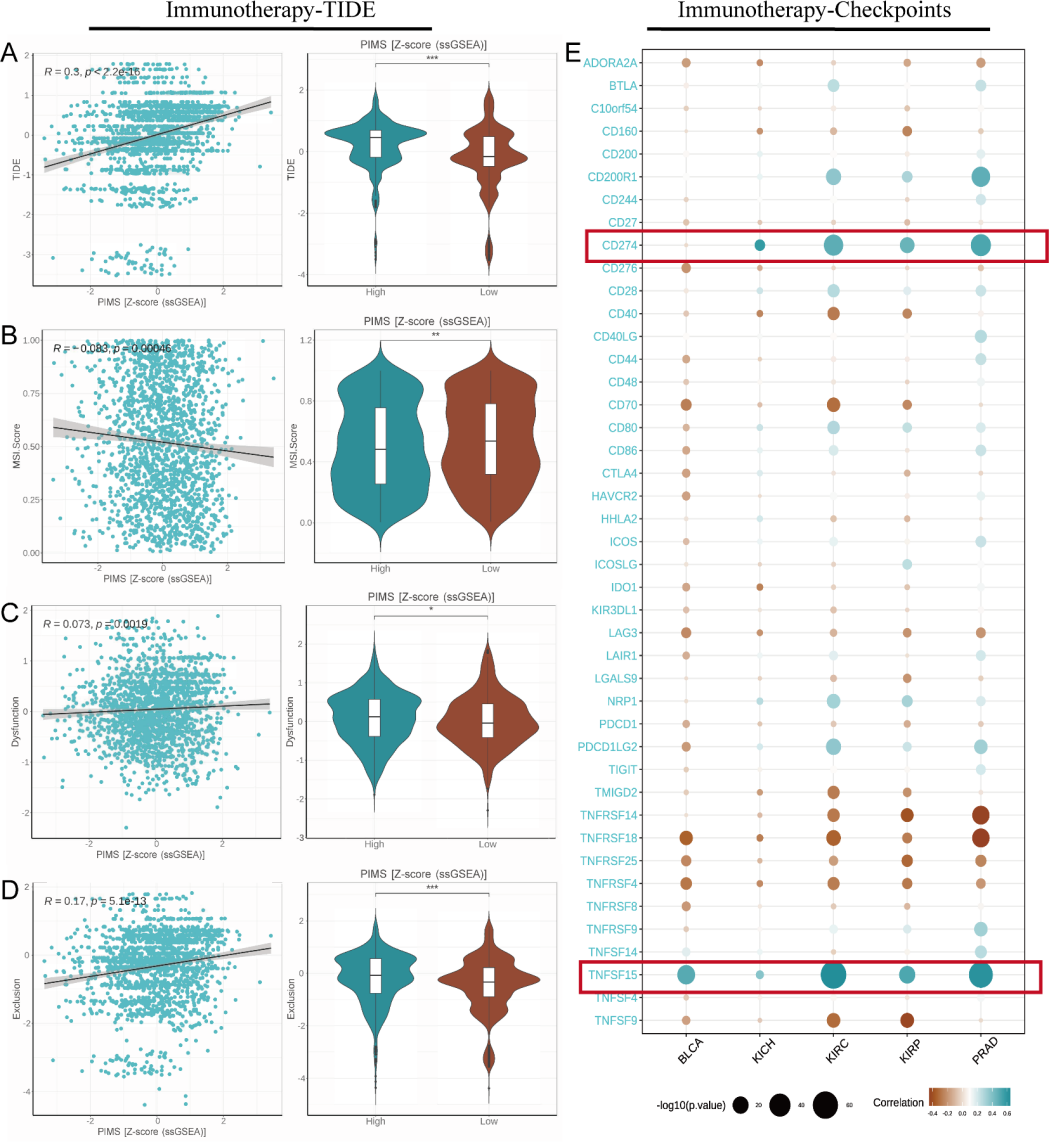
Correlation between PIMS and immune evasion and immune checkpoint expression. (**A**) A significant positive correlation between the PIMS and TIDE score (R = 0.3, *p* < 2.2e−16) is shown in the left panel scatter plot. The violin plot on the right compares TIDE scores between high and low PIMS groups, where higher PIMS correlates with increased immune escape. (**B**–**D**) Weak negative correlations between PIMS and MSI score, immune dysfunction, and immune exclusion were demonstrated. The violin plots showed scores in high versus low PIMS groups, with a small but significant difference. (**E**) The bubble plot demonstrates significant correlations between PIMS and the expression of various immune checkpoint genes across five tumor types, including BLCA, KICH, KIRC, KIRP, and PRAD. The color intensity and size of each bubble represent the strength of correlation and *p* value significance, respectively.

The analysis demonstrated a significant positive correlation between PIMS and the TIDE score (R = 0.3, *p* < 2.2e−16), suggesting that higher PIMS levels are associated with increased immune evasion (Fig. 5A). In contrast, weak negative correlations were observed with MSI (R = 0.083, *p* = 0.00046), dysfunction (R = 0.073, *p* = 0.0019), and immune exclusion (R = 0.17, *p* = 5.1e−13), indicating limited associations between PIMS and microsatellite instability, immune response dysfunction, and an immune-excluding tumor microenvironment (Fig. 5B–D). In the immune checkpoint analysis, CD274 and TNFRSF15 emerged as key checkpoints with significant positive correlations, implying that PIMS may promote or sustain the activity of these inhibitory signals (Fig. 5E). Further analysis revealed that high-PIMS KIRC patients had elevated tumor mutational burden (TMB), suggesting that these patients might respond better to immune checkpoint inhibitors (Supplementary Fig. S5). Investigations into tumor stemness revealed a significant negative correlation between PIMS and stemness markers, where lower PIMS were consistently linked to increased tumor stemness, including DNAss (R = − 0.23, *p* < 2.2e−16) and RNAss (R = − 0.38, *p* < 2.2e−16). In summary, higher PIMS are associated with enhanced immune evasion and a potential for better response to immune checkpoint inhibitors.

### Drug sensitivity analysis

**Fig. 6:**
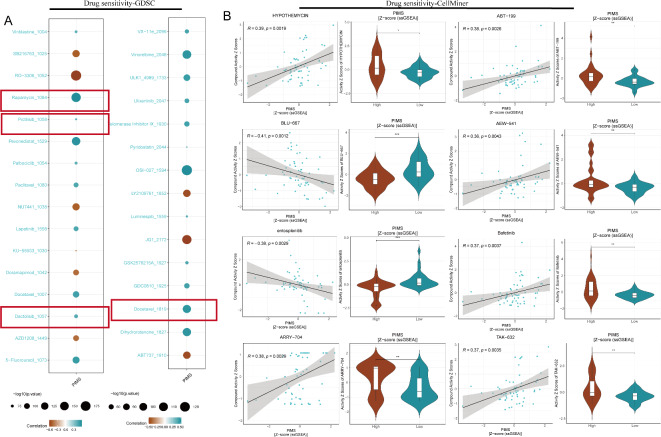
Drug sensitivity and correlation analysis with PIMS across multiple datasets. (**A**) Bubble plot illustrating the correlation between PIMS and IC50 values of anticancer drugs in the GDSC database. The size of the bubbles represents the statistical significance (− log10 *p* value), while the color gradient indicates the strength of the correlation (blue for positive, yellow for negative). (**B**) Scatter plots and violin plots from the CellMiner analysis display significant correlations between PIMS and drug sensitivity scores (standardized z scores). The grey line in scatter plots represents the regression line, while the shaded region shows the confidence interval. The violin plots on the right show the distribution of IC50 values in high- and low-PIMS groups, where the dark lines in the center represent the median.

In the GDSC analysis, several anticancer agents demonstrated significant correlations with drug sensitivity (IC50 values). Positive correlations were observed with traditional targeted drugs (Rapamycin and Docetaxel) as well as PI3K inhibitors (Pictilisi and Dactolisib), indicating that higher PIMS may be linked to increased IC50 values of these agents (Fig. 6A). Similarly, the CellMiner analysis revealed negative correlations with sensitivity scores (Z scores) to BLU-667 and Entosplenitib, while positive correlations were identified for several other agents, including Hypothemycin, ARRY-704, ABT-199, AEW-541, Bafetinib, and TAK-632 (Fig. 6B). This means that BLU-667 and Entosplenitib may be less efficacious in patients in the high-PIMS group. Differential analysis between high- and low-PIMS groups reinforced these findings, as the trends in drug response mirrored those observed in the correlation analyses. This suggests that PIMS may serve as a valuable predictive biomarker for guiding therapeutic strategies, providing insight into potential drug responsiveness based on PIMS levels.

### The establishment and further application of prognostic signature

**Fig. 7:**
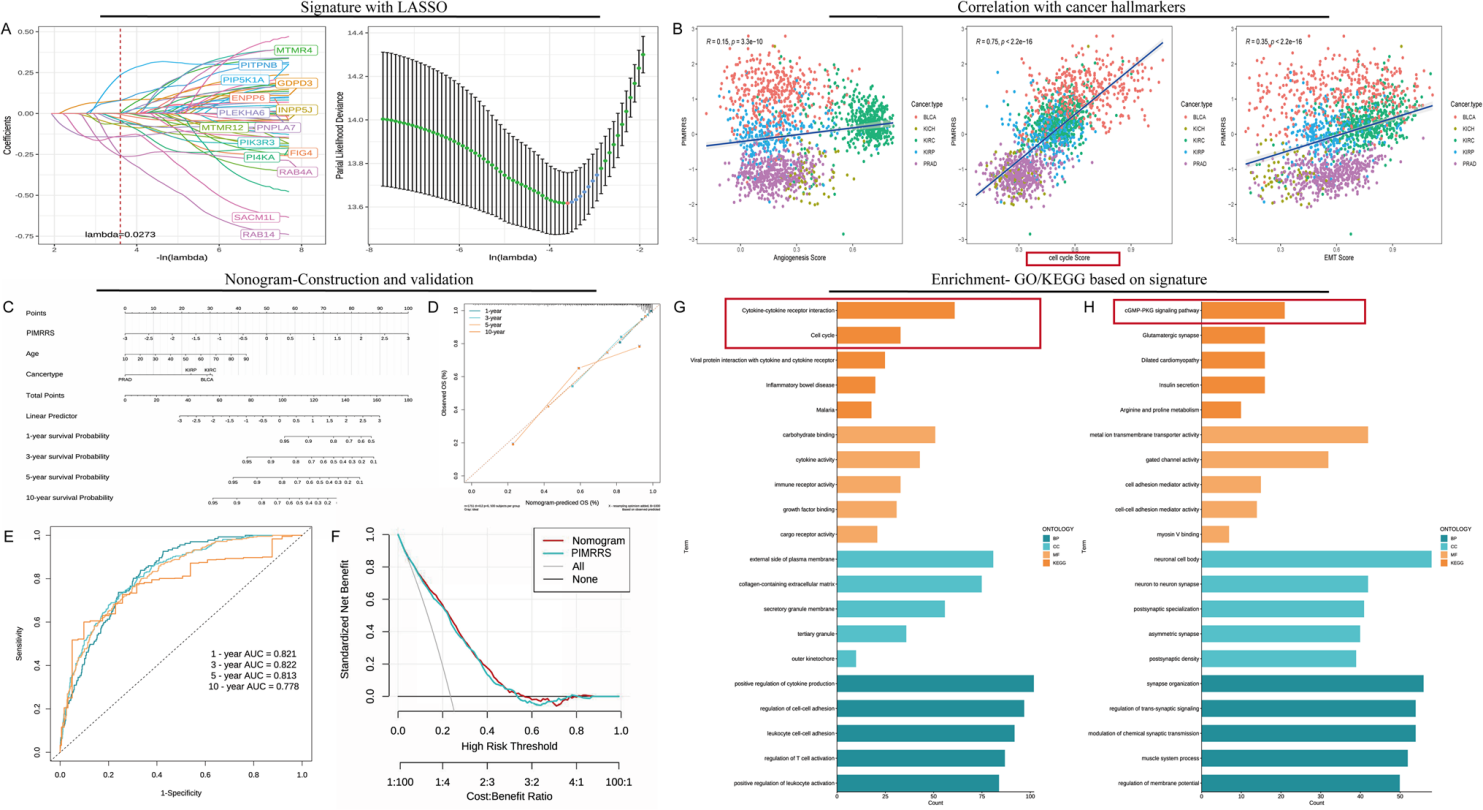
PIMRRS construction, validation, and further application. (**A**) LASSO and Cox regression analysis in the training cohort identified the PI metabolism-related Risk Stratification (PIMRRS) composed of multiple genes, visualized against log(lambda) values. (**B**) Scatter plots represent the significant correlation of PIMRRS with key cancer hallmarks, including angiogenesis, EMT, and cell cycle. (**C**) Nomogram integrating PIMRRS with clinical factors like age and cancer type to predict survival outcomes. (**D**) Calibration plot showing good agreement between predicted and observed survival probabilities. (**E**) ROC curves for predicting 1-, 3-, 5-, and 10-year survival, with AUC values ranging from 0.778 to 0.822. (**F**) Decision curve analysis illustrating the clinical utility of the nomogram. (**G**,**H**) Bar plots of GO and KEGG pathway enrichment analyses show significantly regulated pathways using both up-and down-regulated gene sets.

LASSO and Cox regression analyses were applied to the training cohort, identifying the PIMRRS, which consists of multiple genes contributing variably to the model, as shown by their coefficients plotted against the log(lambda) scale (Fig. 7A). Notably, signature genes such as PIP5K1A and GDPD3 were classified as risk factors, while other genes served as protective elements (Supplementary Fig. S6). The performance of PIMRRS was further evaluated, revealing that patients with BLCA, KIRC, and KIRP had higher PIMRRS scores. PIMRRS was a reliable predictor of survival in all urological cancers, including OS, DSS, and PFI, except OS and DSS in PRAD. KM analysis supported the prognostic relevance of PIMRRS, with patients exhibiting higher PIMRRS scores consistently experiencing worse clinical outcomes. Correlation analysis between PIMRRS and classical cancer hallmarks uncovered significant associations with angiogenesis, cell cycle progression, and EMT, with correlation coefficients of R = 0.15, R = 0.75, and R = 0.35, respectively (*p* < 0.001) (Fig. 7B). These findings further emphasize the prognostic value of PIMRRS in predicting clinical outcomes and its potential links to the cell cycle pathway.

To further strengthen the application in urological cancers, a nomogram incorporating PIMRRS and clinical factors such as age and cancer type was developed, offering a comprehensive tool for survival prediction (Fig. 7C). Calibration plots showed a strong alignment between the nomogram’s predicted outcomes and actual survival rates (Fig. 7D). The nomogram’s predictive accuracy was further validated by ROC curves, with AUC values of 0.821, 0.822, 0.813, and 0.77 for 1-, 3-, 5-, and 10-year survival predictions, respectively, indicating high predictive performance (Fig. 7E). DCA highlighted the nomogram’s clinical utility, demonstrating a superior net benefit over a wide range of decision thresholds (Fig. 7F). GO and KEGG analyses identified significant biological processes and pathways. Enrichment analysis revealed upregulated pathways associated with cytokine-cytokine receptor interaction and cell cycle regulation. Conversely, downregulated genes were primarily linked to cGMP-PKG signaling and glutamatergic synapse pathways (Fig. 7G,H). These findings suggest that the nomogram is a reliable tool for survival prediction when applied to patients with urological cancers.

### Identification and multi-omics landscape of significant genes in PI metabolism

**Fig. 8:**
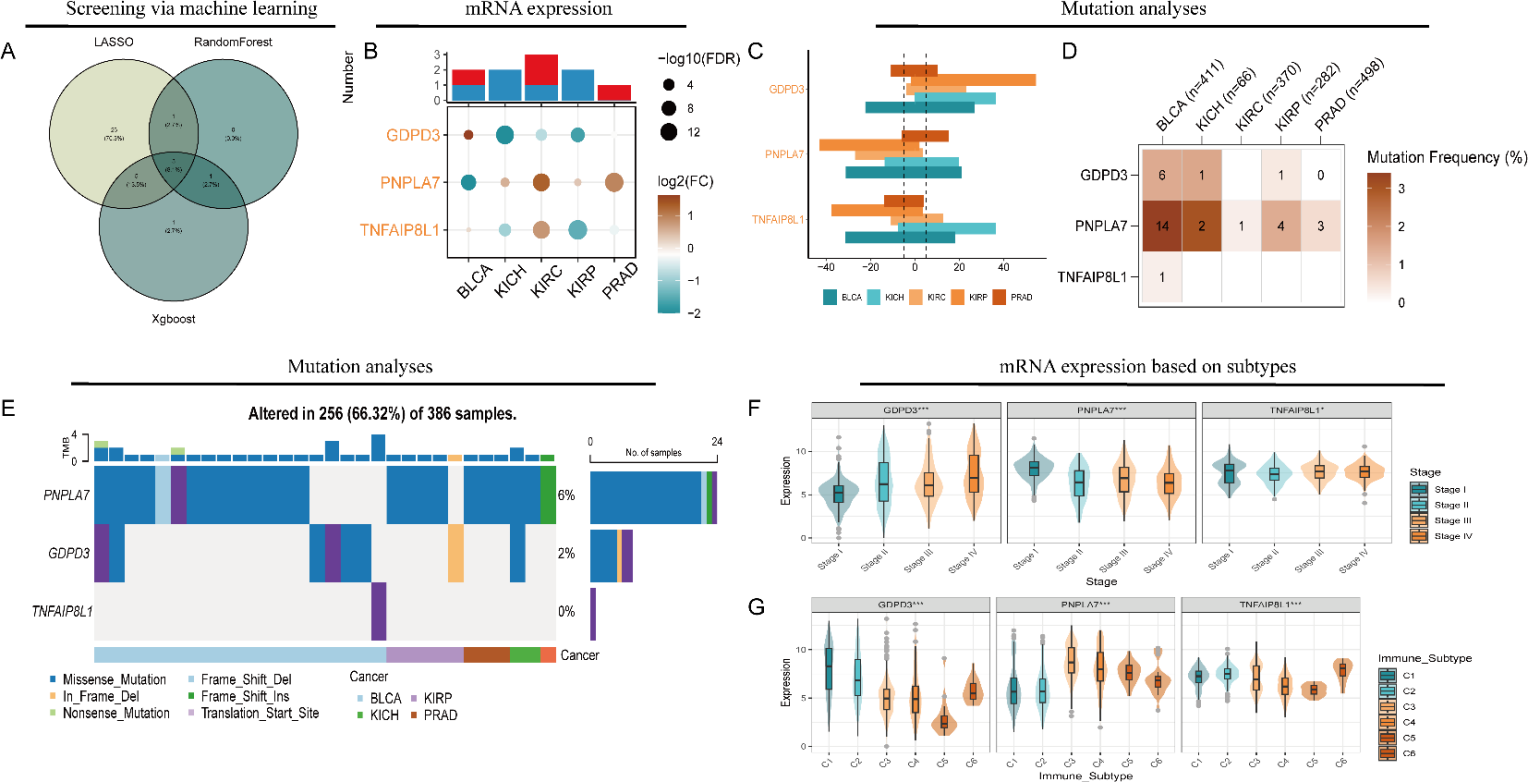
The multi-omics landscape of key genes across urinary cancers. (**A**) Venn diagram showing three intersecting genes (GDPD3, PNPLA7, TNFAIP8L1) identified by LASSO, Random Forest, and XGBoost models. (**B**) Differential expression of these genes in five cancers, where orange shades indicate higher expression and blue denotes lower expression. (**C**) The CNV landscape of key genes in five cancers. (**D**) Mutation frequency heatmap of Single nucleotide variation (SNV) across cancers. (**E**) SNV profiles across samples predominantly show missense mutations of PNPLA7. (**F**) Gene expression violin plots stratified by cancer stage, showing significant upregulation of GDPD3 and downregulation of PNPLA7 with tumor progression. (**G**) Violin plots by immune subtype show distinct expression levels of the three genes across immune clusters.

**Fig. 9:**
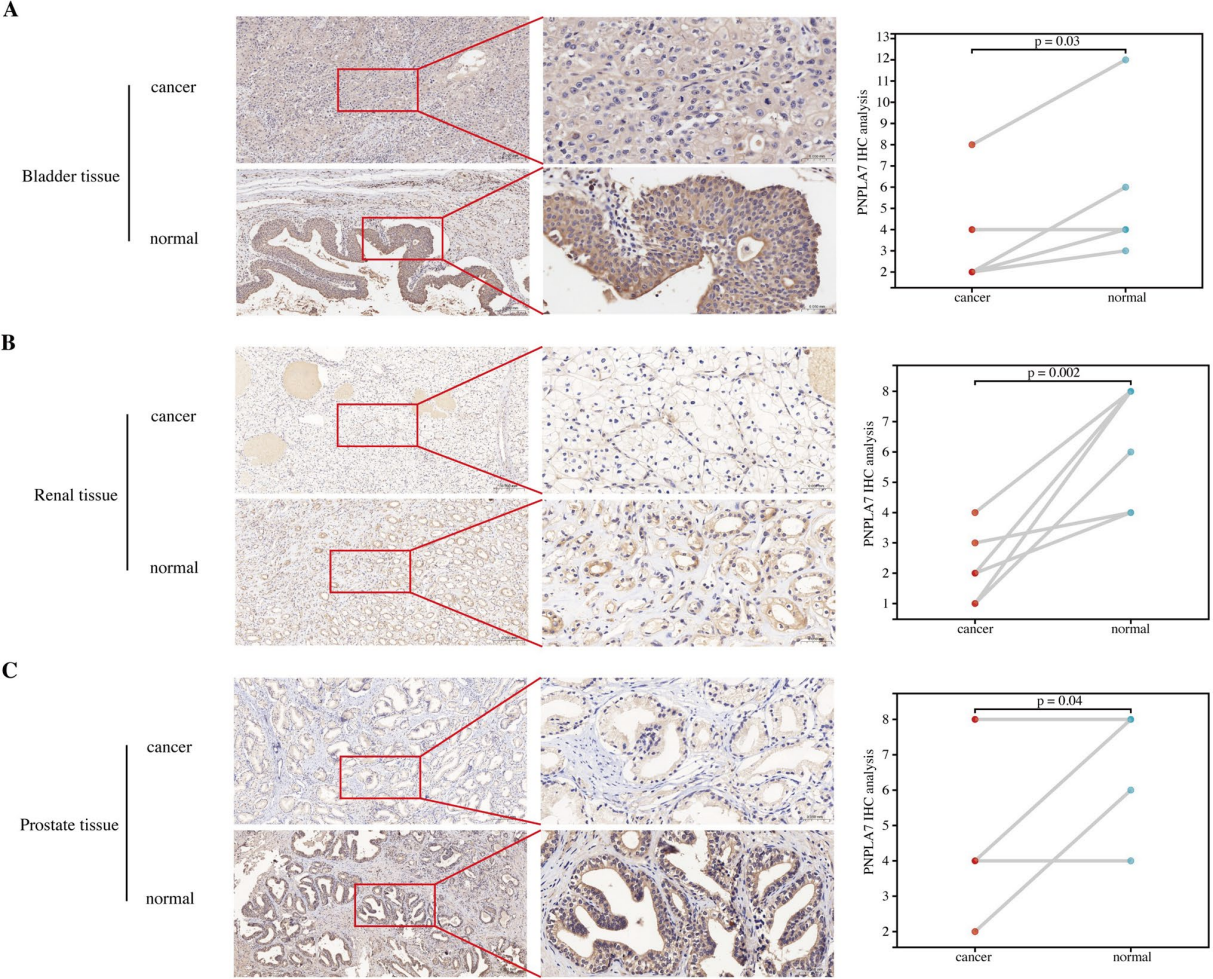
Immunohistochemical (IHC) validation of PNPLA7 expression. (**A**) Bladder cancer IHC results show significantly lower PNPLA7 staining in tumor samples compared to normal tissues (*p* = 0.03). (**B**) Kidney cancer samples show a significant decrease in PNPLA7 expression in cancerous tissue compared to adjacent normal tissues (*p* = 0.002). (**C**) Prostate cancer samples display a similar trend, with lower PNPLA7 levels in cancerous tissues compared to normal tissues (*p* = 0.04). Quantitative analysis of IHC results (right panels) shows a consistent trend of reduced PNPLA7 expression in tumor tissues across all cancer types.

The intersecting genes identified as relevant to patient outcomes from three machine learning methods included GDPD3, PNPLA7, and TNFAIP8L1 (Supplementary Fig. S7 and Fig. 8A). Among these, PNPLA7 achieved the highest score in both the Random Survival Forest (RSF) and XGBoost models. At the RNA level, PNPLA7 was primarily upregulated in KIRC and PRAD, while it was downregulated in BLCA (Fig. 8B). Figure 8C also illustrates the CNV of GDPD3, PNPLA7, and TNFAIP8L1, revealing gains and losses in various urinary cancers. Additionally, SNV analysis showed that PNPLA7 had a high mutation frequency, particularly in BLCA (Fig. 8D), with missense mutations being the predominant mutation type (Fig. 8E). Further investigation revealed that GDPD3 expression increased with advancing clinical stages, while PNPLA7 displayed the opposite trend (Fig. 8F). GDPD3 was more highly expressed in the C1 immune subtype, which may indicate a role in wound healing, whereas PNPLA7 was predominantly expressed in the C3 subtype, suggesting a potential immunoreactive profile (Fig. 8G). Finally, tissue-level analysis of patient samples demonstrated that PNPLA7 was expressed at lower levels in bladder, kidney, and prostate cancers compared to adjacent normal tissues (all *p* < 0.05, Fig. 9A–C), further supporting its relevance in these malignancies.

## Discussion

As mentioned earlier, the incidence and treatment of urological cancers have challenged us in terms of incidence and treatment. And our existing studies on PI metabolism in related cancers are relatively limited, mainly focusing on PI3K/AKT pathways. Research has demonstrated that PI3Ks and their downstream effectors, such as AKT and the mammalian target of rapamycin (mTOR), are critical regulators of glycolysis, cancer metabolism, and cancer cell proliferation^28^. In bladder cancer, the PI3K/AKT signaling pathway has been identified as a key therapeutic target, offering a potential mechanism for treatment^29^. Additionally, in clear cell renal cell carcinoma, hsa_circ_0003596 has been shown to promote proliferation, infiltration, and migration of cancer cells through the miR-502-5p/IGF1R/PI3K/AKT axis^30^. However, the levels of PI metabolism and other PIKGs have not been carefully studied in urological cancers. In conclusion, understanding the complex regulatory network of PI metabolism and its associated signaling pathways is crucial for elucidating the pathogenesis of urological cancers.

Most analyses in this study were conducted at both the pan-urological tumor level and the single tumor level, providing mutual validation and complementarity. Clustering analysis based on PI metabolic pathways revealed that PI metabolism distinctly stratifies patients into two clusters, characterized by marked differences in immune microenvironment, immune checkpoints, and key oncogenic pathways. This underscores the robust clustering capability of PI metabolism. Moreover, significant differences in survival outcomes were observed between the clusters, drawing attention to the potential role of PI metabolism in modulating survival. Consequently, we further investigated the expression profiles of PI metabolism through bulk sequencing and single-cell analyses of urological tumors, the multi-omics landscape of survival-related PIKGs, and the interplay between PI metabolism and the immune microenvironment, particularly in single-cell contexts.

To investigate the expression pattern of PI metabolism, we employed algorithms to assess PIMS in transcriptomes derived from both bulk and single-cell sequencing data. At the tissue level, PIMS was found to be reduced in tumor tissues and decreased progressively with disease advancement, illustrating a clear association between PI metabolism activity and tumor progression, where lower PIMS reflects more advanced disease. Further examination at the cellular level within the single-cell dataset revealed significant heterogeneity in PIMS, with pronounced variations across cell types in different tumors. Notably, cancer cells in KIRC exhibited the lowest PIMS and had greater interactions with immune and stromal cells, particularly through CD70 and EDN pathway networks. Conversely, cancer cells in BLCA and PRAD showed higher PIMS and those with elevated PIMS demonstrated increased intercellular associations, as evidenced by cell–cell communication analyses. Previous studies have underscored the importance of these pathway networks in modulating tumor-immune interactions. For instance, PTN regulates endothelial cell activation and angiogenesis via its receptor PTPRZ1^31^. Additionally, the interaction of CD70 with its receptor CD27 enhances T-cell proliferation, differentiation, and immune response^32^. In summary, PIMS expression exhibits both commonalities and substantial heterogeneity, spanning from tissue to cellular levels and across different tumor subtypes.

Another notable finding is the impact of PIMS on survival outcomes in patients with urological tumors. Prognostic analysis of the TCGA dataset initially demonstrated a significant association between transcriptome-level PIMS and clinical outcomes, showing that patients with lower PIMS exhibited shorter survival times. In diagnostics, PIMS provides a quantitative measure based on mRNA expression levels, supporting early and accurate tumor detection. With sufficient validation, it could establish diagnostic thresholds, enhancing its utility in distinguishing normal from pathological conditions. These diagnostic insights can further guide treatment strategies. For example, PIMS’s ability to reflect immune-related tumor characteristics may help identify patients likely to benefit from immune checkpoint inhibitors or specific chemotherapies, enabling clinicians to tailor therapies and improve therapeutic efficacy. In prognosis, PIMS plays a central role in the PIMRRS model, stratifying patients into risk categories for refined outcome prediction. Combining PIMRRS with other clinical parameters into a nomogram could further improve prognostic accuracy, supporting personalized follow-up plans and long-term management. Despite its potential, PIMS faces challenges such as the need for extensive multi-center validation and the development of cost-effective, standardized assays for clinical use. Future research should prioritize these validations while integrating PIMS with multi-omics approaches to expand its utility across diverse tumor types. By addressing these challenges, PIMS could significantly enhance clinical decision-making, optimizing both diagnostic and therapeutic outcomes.

Additionally, machine learning techniques such as LASSO regression, XGBoost, and random forest were applied to refine the results of PIMS and identify the three PIKGs most relevant to prognosis. These algorithms were chosen due to their unique strengths in handling high-dimensional data and feature selection. LASSO is highly effective at inducing sparsity and selecting key features while mitigating overfitting. RSF, with its ensemble-based approach, excels in capturing complex variable interactions and nonlinear relationships. XGBoost, renowned for its computational efficiency and strong predictive performance, further enhances the analysis. Compared to previous studies^33,34^, such as stepwise Cox regression, these approaches provide greater robustness and adaptability. By integrating their outputs, we reduced the biases inherent to any single algorithm, leveraging their complementary strengths to enhance reliability and confidence in identifying the final list of prognostic genes. These genes were then explored from multiple angles, including expression profiles, CNV, and SNV correlations, revealing intricate interrelationships. Among these analyses, the gene PNPLA7 with the best performance in machine learning was selected for further IHC validation. And it was exactly downregulated in the BLCA, KIRC, and PRAD. Zhang et al. had previously found that PNPLA7 was deregulated in liver cancer due to hypermethylation^35^. Recently, PNPLA7 was also found to be lowly expressed and act as a biomarker in gastric and colorectal cancer^36^. Combined with our findings, PNPLA7 is likely to be a cancer suppressor gene that acts as a promising biomarker in various tumors. This work not only enhances our understanding of how PI metabolism influences patient prognosis but also enables more accurate prognostic predictions, providing new insights for clinical practice.

The relationship between PI metabolism and the immune microenvironment represents another crucial aspect of this study. Using algorithms to assess immune cell infiltration in transcriptome analyses at the tissue level, we observed a strong correlation between PI metabolism and the immune microenvironment, with a particularly stable and high correlation to M2 macrophages across both the overall cohort and sub-tumor analyses. Previous studies have identified genes such as lncRNA AGAP2-AS1 and mRNA CNTNAP1, which influence M2 macrophage polarization^37,38^, subsequently impacting the tumor microenvironment and progression. Additionally, extensive research has shown that lipid metabolism can modulate macrophages behavior, further affecting tumor progression. Wang et al. demonstrated that polyunsaturated fatty acids promote M2-like tumor-associated macrophage infiltration in the ovarian cancer microenvironment by inhibiting the RhoA-YAP1 signaling pathway^39^. Zhou et al. further revealed that short-chain fatty acids encourage M2-like macrophage deposition by enhancing M1 polarization in the tumor microenvironment, reversing glioblastoma progression associated with intestinal dysbiosis^40^. Collectively, our findings provide a novel promising insight that we may affect the M2 macrophage infiltration and tumor progress via manipulating PI metabolism.

This study provides a comprehensive multi-omics and multi-level analysis of the role of PI metabolism in urologic tumors, with the integration of machine learning and experimental validation being key highlights. However, several aspects warrant further investigation. A potential limitation of this study is the relatively small number of tumor samples used for IHC validation. Further large-scale, multi-center validations may help us to gain more insight into our findings. Second, PI metabolism in this study was assessed based on pathway-related genes without quantifying relevant exact metabolite changes, which is an area that could be addressed by metabolomics in future research.

## Electronic supplementary material

Below is the link to the electronic supplementary material.

Supplementary Material 1

## Data Availability

The RNA-sequencing data and corresponding clinical information as well as mutation-related data were downloaded from the Cancer Genome Atlas (TCGA) database (https://portal.gdc.cancer.gov/), the University of California Santa Cruz (UCSC) Genome Browser website (https://genome.ucsc.edu/). The single-cell RNA sequencing data GSE207493, GSE135337, and GSE141445 were collected from Gene Expression Omnibus (GEO) database.
