# Supplementary material for: Comprehensive bioinformatics and immunohistochemical analyses identify phosphoinositide metabolism and PNPLA7 as potential biomarkers in urological cancers
Source: Sci Rep. 2025 Apr 12;15:12588. doi: 10.1038/s41598-025-97118-9 (PMC11993629; doi:10.1038/s41598-025-97118-9)

**Supplementary Figs. S1-S7**

**Supplementary Fig. S1: Single-cell analysis from BLCA shows PIMS-based pathway networks.**

(A) UMAP plot shows the clustering of cellular populations, with distinct groups labeled by cell type. (B) The bubble plot displays feature expression, with bubble size representing the proportion of cells expressing a given marker and color intensity representing average expression levels. Key markers are shown for each cell type. (C) Violin plot of PIMS across cell types demonstrates variability in score distribution. (D) Sankey diagrams illustrate the outgoing and incoming communication patterns between high-PIMS cancer cells, low-PIMS cancer cells, B cells, and fibroblasts. (E) The dot plot shows the contribution of each cell type to outgoing and incoming communication patterns.


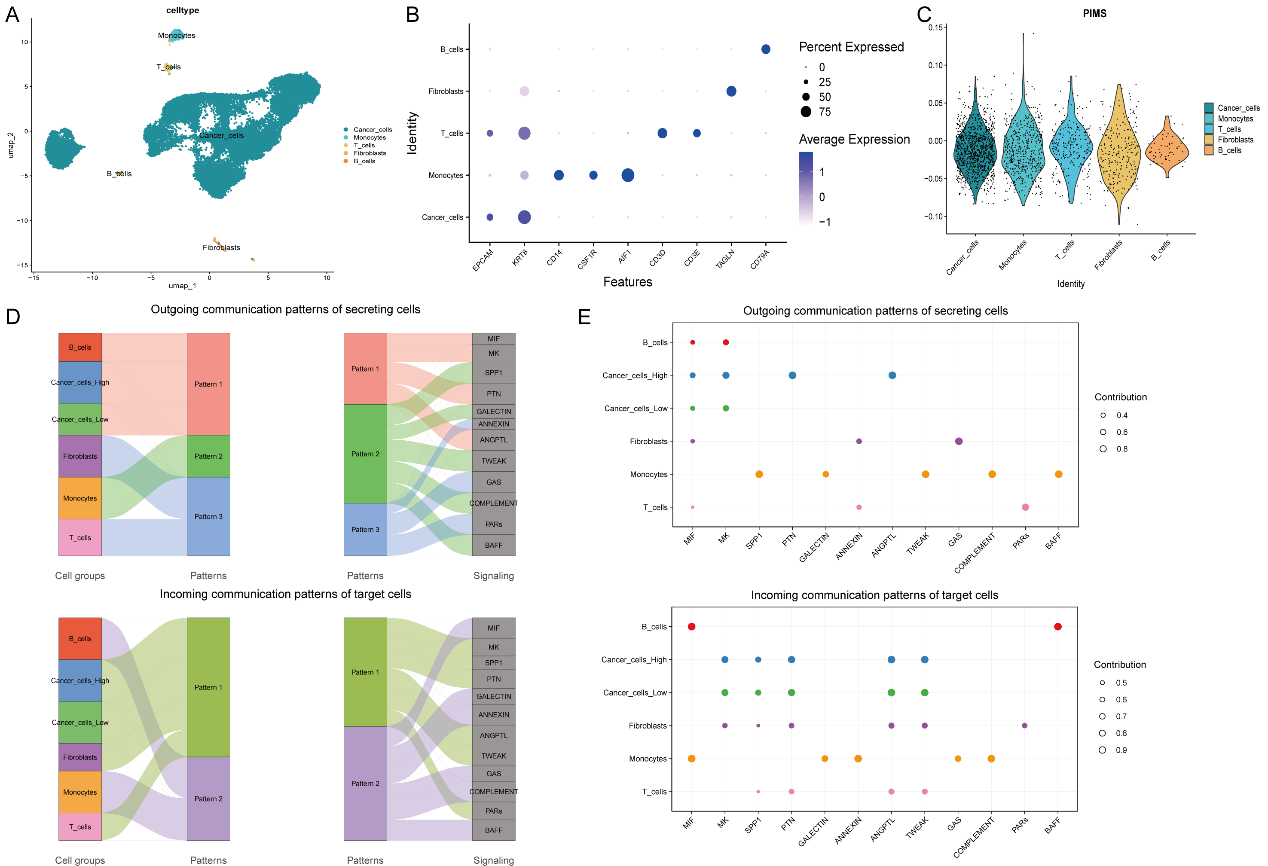


**Supplementary Fig. S2: Single-cell analysis from KIRC reveals PIMS-based pathway interactions.**

(A) UMAP plot shows cell clustering in KIRC, with distinct cell populations. (B) Bubble plot indicates feature expression across cell types, with the size of the bubble representing the percent of cells expressing a marker and color intensity representing average expression. (C) Violin plot displays the distribution of PIMS scores across cell types. (D) Sankey diagram illustrates outgoing communication patterns from high- and low-PIMS cancer cells, highlighting their interaction with fibroblasts, myeloid cells, and T cells. (E) The dot plot presents the contribution of each cell type to outgoing and incoming communications.


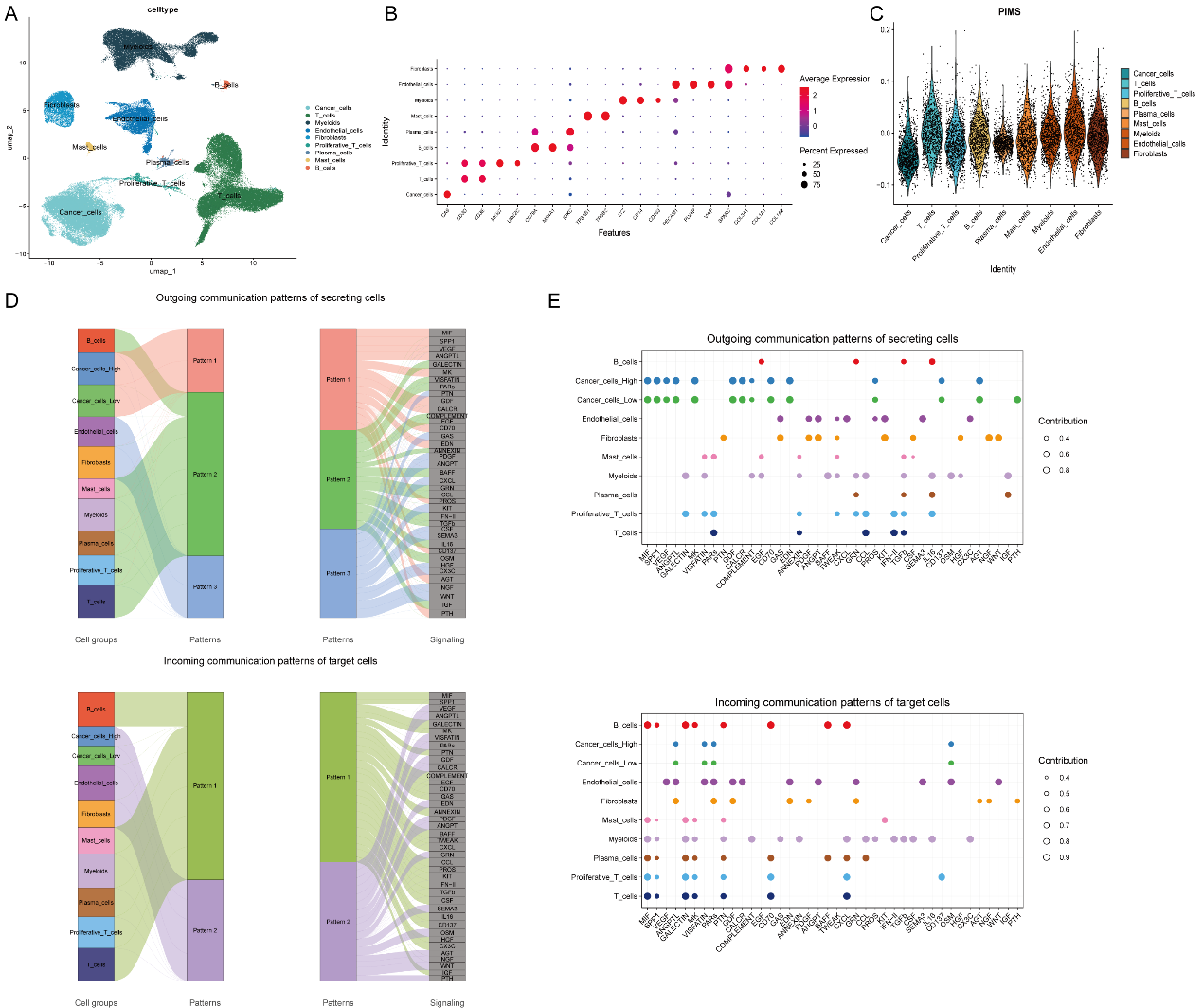


**Supplementary Fig. S3: Single-cell analysis from PRAD highlights PIMS-based pathway communication.**

(A) UMAP plot of PRAD cell types, showing distinct clusters of cancer cells, fibroblasts, myeloid cells, and stromal cells. (B) The bubble plot presents feature expression across cell types, with markers and features identified for major populations. (C) Violin plots of PIMS across different cell types. (D) Sankey diagrams highlight outgoing and incoming communication patterns for high- and low-PIMS cancer cells. (E) The dot plots of outgoing and incoming communication patterns.


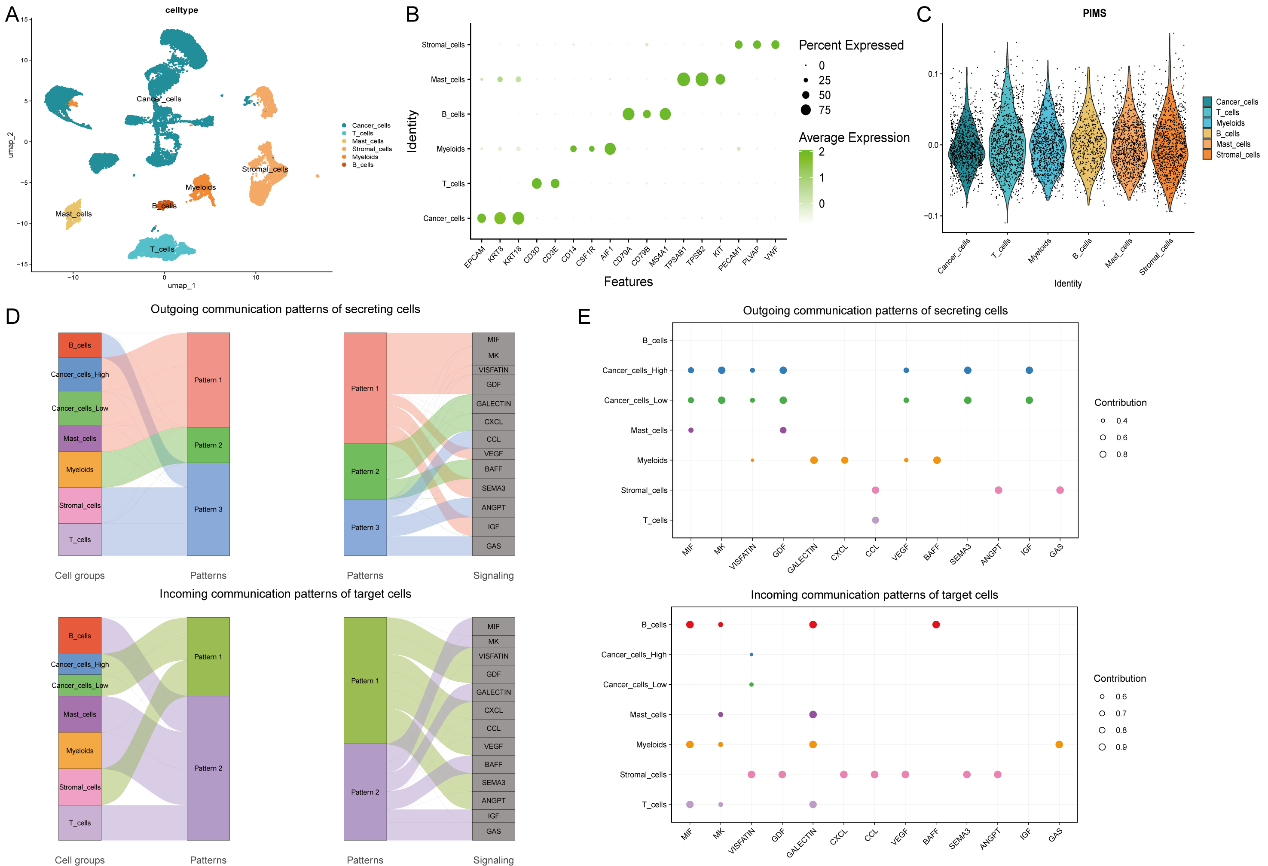


**Supplementary Fig. S4: Tumor microenvironment scores and immune infiltration in relation to PIMS.**

(A) Boxplots display the distribution of StromalScore, ImmuneScore, and EstimateScore in five different cancer types. High-PIMS patients in KIRC and PRAD demonstrate significantly higher scores compared to their low-PIMS counterparts. Conversely, in BLCA, lower scores are observed in the high-PIMS group. Significance levels are denoted: ns (non-significant), * (p < 0.05), ** (p < 0.01), *** (p < 0.001), **** (p < 0.0001). (B) Correlation scatter plots between PIMS and StromalScore, ImmuneScore, as well as EstimateScore. The colored dots represent cancer types.


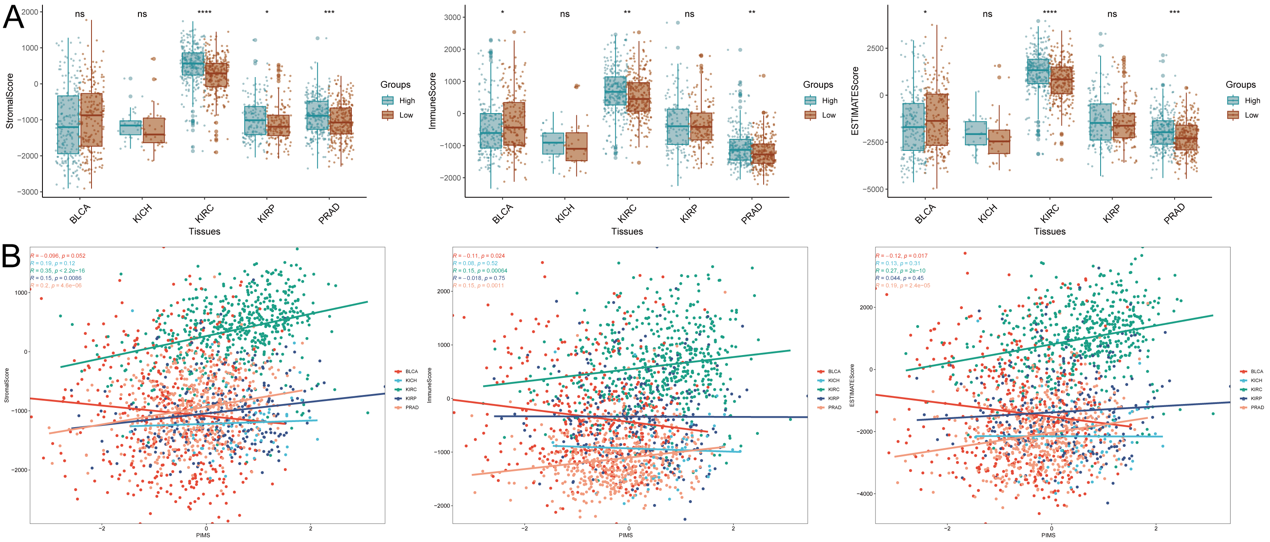


**Supplementary Fig. S5: Tumor mutational burden (TMB) and stemness association with PIMS.**

(A) Boxplots show the comparison of TMB between high and low PIMS groups across five tumor type. A significant increase in TMB is noted in high-PIMS KIRC patients (p < 0.0001), as indicated by ****, while other cancer types exhibit no significant difference. The central line in each box represents the median. (B) DNAss (top) and RNAss (bottom) show strong negative correlations with PIMS (R = -0.23 and R = -0.38, respectively, both p < 2.2e-16), suggesting that lower PIMS is associated with higher tumor stemness. Violin plots display DNAss and RNAss scores in high and low PIMS groups, confirming the inverse relationship between PIMS and tumor stemness.


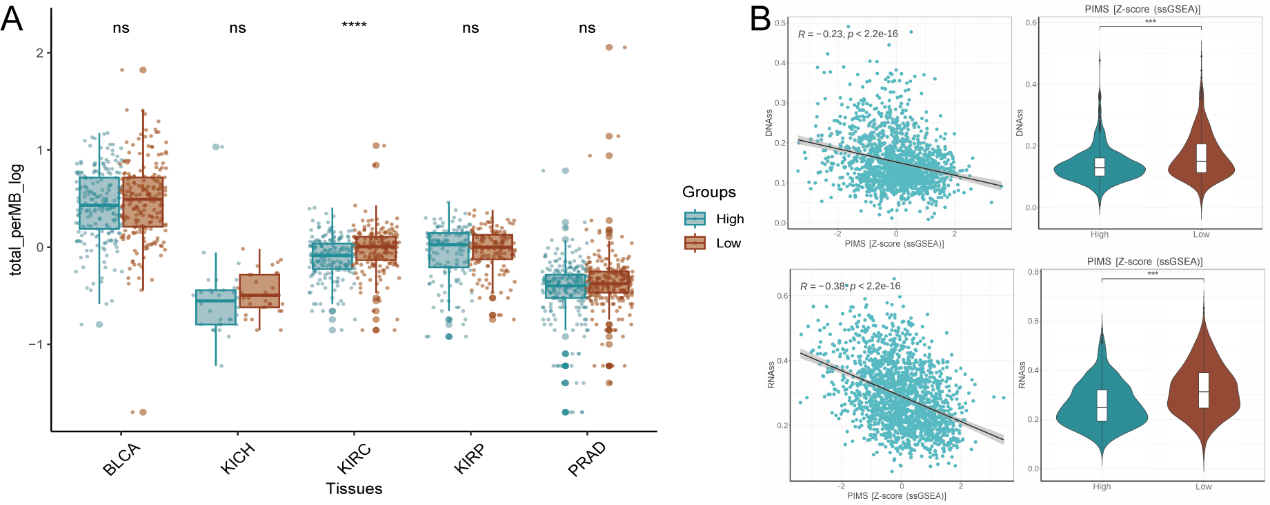


**Supplementary Fig. S6: Components and initial validation of PIMRRS.**

(A) Forest plot of hazard ratios (HR) for selected genes shows risk (HR > 1) and protective (HR < 1) factors. (B) Violin plots illustrate the distribution of PIMRRS across different patient subgroups, and the dot plot shows the prognostic value of PIMRRS. Dot sizes reflect the significant level. (C) Kaplan-Meier survival curves for OS, DSS, and PFI in high versus low PIMRRS groups across various groups.

**
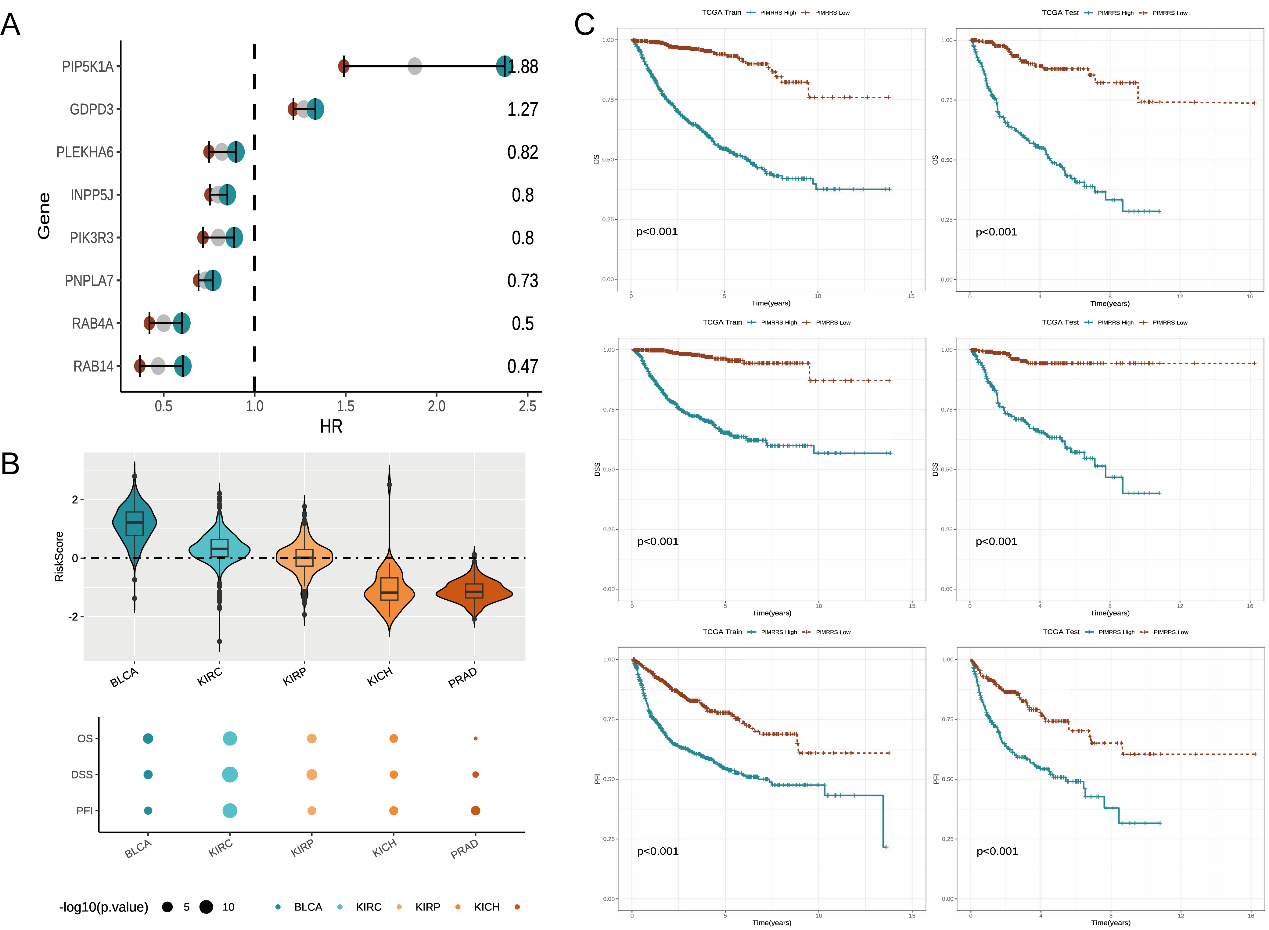
**

**Supplementary Fig. S7: Machine learning-based gene screening.**

(A) Error rate comparison across 1,000 trees for Random Survival Forest (RSF). The decreasing curve indicates increasing model accuracy as more trees are added. (B) Cross-validation curve for the LASSO regression model (top) and the progression of the coefficients for selected genes (bottom). (C) The negative log-likelihood (val2_cox_nloglik) decreases with increasing iterations of the XGBoost model, indicating an improvement in model performance on the validation set. (D) Feature importance ranked by gain in XGBoost. Blue bars represent the magnitude of each feature’s contribution to the model.


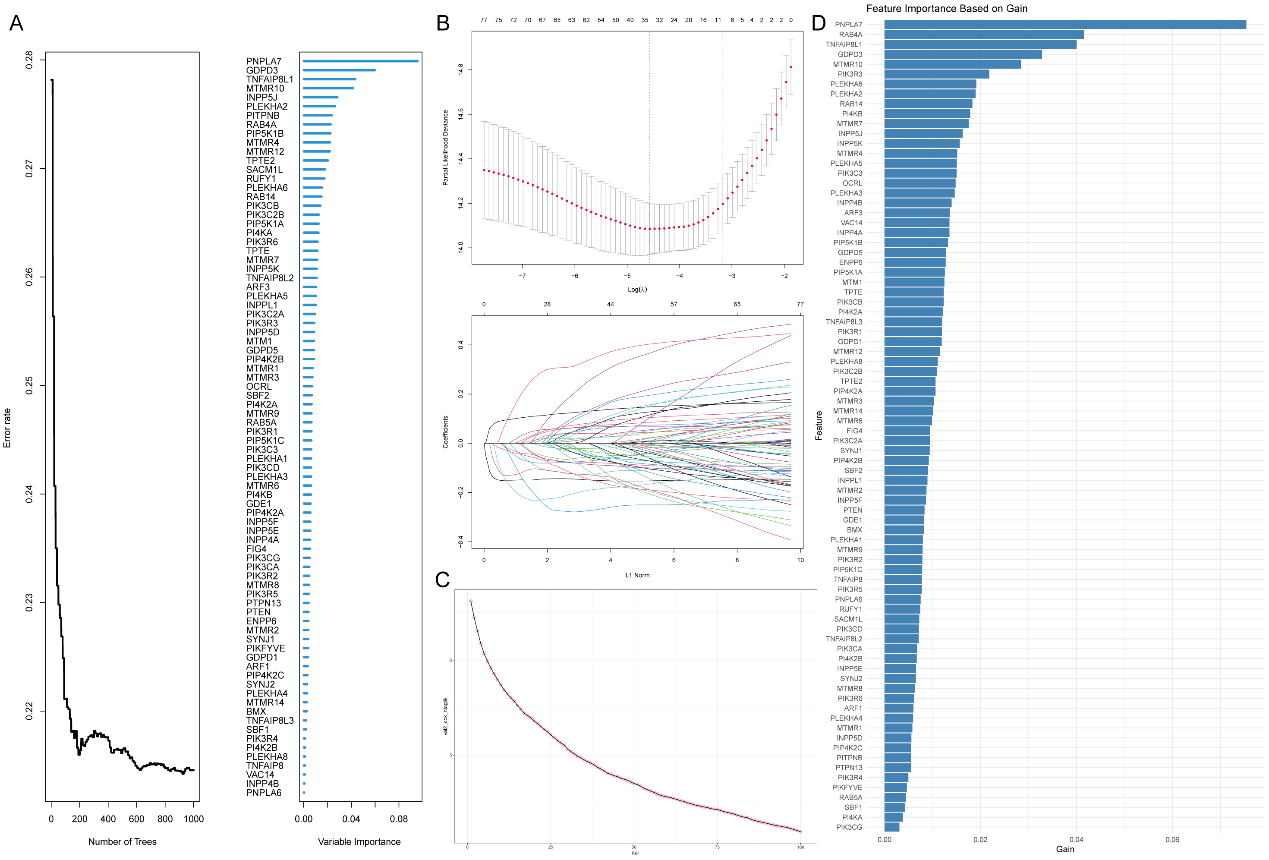

Supplement: Supplementary file 1 — Supplementary Material 1 [file 41598_2025_97118_MOESM1_ESM.docx]
